# Retraction Note: One-step assembly of 2H-1T MoS_2_:Cu/reduced graphene oxide nanosheets for highly efficient hydrogen evolution

**DOI:** 10.1038/s41598-022-17706-x

**Published:** 2022-08-02

**Authors:** H.-Y. He

**Affiliations:** College of Material Science and Engineering, Shaanxi University of Science and Technology, Weiyang, 710021 China

Retraction of: *Scientific Reports* 10.1038/srep45608, published online 13 April 2017

Editors have retracted this Article.

After publication of this paper concerns were raised about unusually high level of similarity in the background noise for two different samples in Figure 3B, as well as for two sets of samples in Figure 3A. The Author was able to provide data for this figure, but these did not resolve the Editors' concerns. The Author is also not able to provide the original data for the other figures. Editors therefore no longer have confidence in the results reported in this study.

H.-Y. He agrees with the retraction and its wording.

